# Supplementary material for: PCR-based detection and phylogenetic analysis of Candidatus Liberibacter asiaticus in citrus orchards across Nepal
Source: PLoS One. 2026 May 27;21(5):e0333726. doi: 10.1371/journal.pone.0333726 (PMC13215486; doi:10.1371/journal.pone.0333726)
Supplement: S1 File — (PDF) [file pone.0333726.s001.pdf]

Sequence data that support the findings of this study have been deposited in the National Center for Biotechnology Information (NCBI) with primary accession code PP916596 to PP916605.

>PP916596.1 Uncultured *Candidatus* Liberibacter sp. clone WTHC_05_Clas_Kathmandu_Nepal_RVS 16S ribosomal RNA gene, partial sequence (>WTHC_05_Clas_Kathmandu_Nepal_RVS)

GAGGGTAACTATCTGCGGGGGGTTGCGCTCGTTGCGGGACTTAACCCAACATCTCACGACACGAGCTG
ACGACAGCCATGCAGCACCTGTGTAAAGGTCTCCGAAAAGAAAATACCATCTCTGATATCGTCCTATACA
TGTCAAGGGCTGGTAAGGTTCTGCGCGTTGCATCGAATTAAACCACATGCTCCACCGCTTGTGCGGGCC
CCCGTCAATTCCTTTGAGTTTTAATCTTGCGACCGTACTCCCCAGGCGGAGTGCTTAATGCGTTAGCTGC
GCCACTGAATGGTAAACCACCCAACAGCTAGCACTCATCGTTTACGGCGTGGACTACCAGGGTATCTAA
TCCTGTTTGCTCCCCACGCTTTCGCGCCTCAGCGTCAGTATCAGGCCAGTGAGCCGCCTTCGCCACCGG
TGTTCCTCCGAATATCTACGAATTTCACCTCTACACTCGGAAT

>PP916597.1 Uncultured *Candidatus* Liberibacter sp. clone WTHC_04_Clas_Dadeldhura_Nepal_RVS 16S ribosomal RNA gene, partial sequence (>WTHC_04_Clas_Dadeldhura_Nepal_RVS)

GAGGTACATGTGTGGGGTTGCGCTCGTTGCGGGACTTAACCCAACATCTCACGACACGAGCTGACGAC
AGCCATGCAGCACCTGTGTAAAGGTCTCCGAAAAGAAAATACCATCTCTGATATCGTCCTATACATGTCA
AGGGCTGGTAAGGTTCTGCGCGTTGCATCGAATTAAACCACATGCTCCACCGCTTGTGCGGGCCCCCGT
CAATTCCTTTGAGTTTTAATCTTGCGACCGTACTCCCCAGGCGGAGTGCTTAATGCGTTAGCTGCGCCAC
TGAATGGTAAACCACCCAACAGCTAGCACTCATCGTTTACGGCGTGGACTACCAGGGTATCTAATCCTGT
TTGCTCCCCACGCTTTCGCGCCTCAGCGTCAGTATCAGGCCAGTGAGCCGCCTTCGCCACCGGTGTTCC
TCCGAATATCTACGAATTTCACCTCTACACTCGGAAT

>PP916598.1 Uncultured *Candidatus* Liberibacter sp. clone WTHC_03_Clas_Dailekh_Nepal_RVS 16S ribosomal RNA gene, partial sequence (>WTHC_03_Clas_Dailekh_Nepal_RVS)

GGTGGTACAGTGTTGGGGGTTGCGCTCGTTGCGGGACTTAACCCAACATCTCACGACACGAGCTGACG
ACAGCCATGCAGCACCTGTGTAAAGGTCTCCGAAAAGAAAATACCATCTCTGATATCGTCCTATACATGT
CAAGGGCTGGTAAGGTTCTGCGCGTTGCATCGAATTAAACCACATGCTCCACCGCTTGTGCGGGCCCCC
GTCAATTCCTTTGAGTTTTAATCTTGCGACCGTACTCCCCAGGCGGAGTGCTTAATGCGTTAGCTGCGCC
ACTGAATGGTAAACCACCCAACAGCTAGCACTCATCGTTTACGGCGTGGACTACCAGGGTATCTAATCCT
GTTTGCTCCCCACGCTTTCGCGCCTCAGCGTCAGTATCAGGCCAGTGAGCCGCCTTCGCCACCGGTGTT
CCTCCGAATATCTACGAATTTCACCTCTACACTCGGAAT

>PP916599.1 Uncultured *Candidatus* Liberibacter sp. clone WTHC_02_Clas_Myagdi_Nepal_RVS 16S ribosomal RNA gene, partial sequence (>WTHC_02_Clas_Myagdi_Nepal_RVS)

GGGGGGACATGTGGGGGGGTTGCGCTCGTTGCGGGACTTAACCCAACATCTCACGACACGAGCTGACG
ACAGCCATGCAGCACCTGTGTAAAGGTCTCCGAAAAGAAAATACCATCTCTGATATCGTCCTATACATGT
CAAGGGCTGGTAAGGTTCTGCGCGTTGCATCGAATTAAACCACATGCTCCACCGCTTGTGCGGGCCCCC
GTCAATTCCTTTGAGTTTTAATCTTGCGACCGTACTCCCCAGGCGGAGTGCTTAATGCGTTAGCTGCGCC
ACTGAATGGTAAACCACCCAACAGCTAGCACTCATCGTTTACGGCGTGGACTACCAGGGTATCTAATCCT
GTTTGCTCCCCACGCTTTCGCGCCTCAGCGTCAGTATCAGGCCAGTGAGCCGCCTTCGCCACCGGTGTT
CCTCCGAATATCTACGAATTTCACCTCTACACTCGGAAT

>PP916600.1 Uncultured *Candidatus* Liberibacter sp. clone WTHC_01_Clas_Palpa_Nepal_RVS 16S ribosomal RNA gene, partial sequence (>WTHC_01_Clas_Palpa_Nepal_RVS)

GGGGGTGTAATATCTGGGGGGGGTTGCGCTCGTTGCGGGACTTAACCCAACATCTCACGACACGAGCTG
ACGACAGCCATGCAGCACCTGTGTAAAGGTCTCCGAAAAGAAAATACCATCTCTGATATCGTCCTATACA
TGTCAAGGGCTGGTAAGGTTCTGCGCGTTGCATCGAATTAAACCACATGCTCCACCGCTTGTGCGGGCC
CCCGTCAATTCCTTTGAGTTTTAATCTTGCGACCGTACTCCCCAGGCGGAGTGCTTAATGCGTTAGCTGC
GCCACTGAATGGTAAACCACCCAACAGCTAGCACTCATCGTTTACGGCGTGGACTACCAGGGTATCTAA

TCCTGTTTGCTCCCCACGCTTTCGCGCCTCAGCGTCAGTATCAGGCCAGTGAGCCGCCTTCGCCACCGG
TGTTCCTCCGAATATCTACGAATTTCACCTCTACACTCGG

>PP916601.1 Uncultured *Candidatus* Liberibacter sp. clone WTHC_05_Clas_Kathmandu_Nepal_FWD 16S ribosomal RNA gene, partial sequence (>WTHC_05_Clas_Kathmandu_Nepal_FWD)

GACTACACCGGTGGCGAAGGTGGCTCACTGGCCTGATACTGACGCTGAGGGGCGAAAGCATGGGGAGC
AAACAGGATTAGATACCCTGGTAGTCCACGCCGTAAACGATGAGTGCTAGCTGTTGGGTGGTTTACCATT
CAGTGGCGCAGCTAACGCATTAAGCACTCCGCCTGGGGAGTACGGCGGCAAGATTAAAACTACAAGGA
ATTGACGGGCTCCCGCACAAGCGGGGGAGCTTGTGTTTTATTTCGATGTGCCGCGCAGAACCTTACCAG
CCCTTGACAGGGTTGGAACGATATCAGAGATGGTATTTTCTTTTCGGAGACCTTACCACAGGTGCTGCAT
GGTGGTCGCCAGCTCGTGTCGTGAGATGTTGGGGTTAAGTCCCGCAACGGAGCGCAACCCCTGCCTCTA
GTTGCCATCAGCCTTTAGCTTTTGACCTAGATGTTGGGTACACATT

>PP916602.1 Uncultured *Candidatus* Liberibacter sp. clone WTHC_04_Clas_Dadeldhura_Nepal_FWD 16S ribosomal RNA gene, partial sequence (>WTHC_04_Clas_Dadeldhura_Nepal_FWD)

AGGTACACCGGCGGCCAAGGCGGCTCACTGGCCTGATACTGACGCTGAGGCGCGAAAGCGTGTGGAGC
AAACAGGATTAGATACCCTGGTAGTCCACGCCGTAAACGATGAGTGCTAGCTGTTGGGTGGTTTACCATT
CAGTGGCGCAGCCAACGCATTAAGCACTCCGCCTGGGGAGTACGGTCGCCAGATTAAAACTCAAAGGA
ATTGACGGGGGCCCGCACAAGCGGTGGAGCATGTGGTTTAATTCGATGCAACGCGCAGAACCTTACCA
GCCCTTGACATGTATAGGACGATATCAGAGATGGGATTTTCTTTTCGGAGACCTTTACACAGGTGCTGCA
TGGCTGTCGTCAGCTCGTGTCGTGAGATGTTGGGTTAAGTCCCGCAACGAGCGCAACCCCTGCCTCTAG
TTGCCATCAAGTTTATTCTTCTACCTAGAAAAAGGGTACCCGACTT

>PP916603.1 Uncultured *Candidatus* Liberibacter sp. clone WTHC_03_Clas_Dailekh_Nepal_FWD 16S ribosomal RNA gene, partial sequence (>WTHC_03_Clas_Dailekh_Nepal_FWD)

AGGTACACCGGTGGCGAAGGCGGCTCACTGGCCTGATACTGACGCTGAGGCGCGAAAGCGTGGGGAG
CAAACAGGATTAGATACCCTGGTAGTCCACGCCGTAAACGATGAGTGCTAGCTGTTGGGTGGTTTACCAT
TCAGTGGCGCAGCTAACGCATTAAGCACTCCGCCTGGGGAGTACGGTCGCAAGATTAAAACTCAAAGG
AATTGACGGGGGCCCGCACAAGCGGTGGAGCATGTGGTTTAATTCGATGCAACGCGCAGAACCTTACC
AGCCCTTGACATGTATAGGACGATATCAGAGATGGTATTTTCTTTTCGGAGACCTTTACACAGGTGCTGC
ATGGCTGTCGTCAGCTCGTGTCGTGAGATGTTGGGTTAAGTCCCGCAACGAGCGCAACCCCTGCCTCTA
GTTGCCATCAAGTTTAGGTTTTAACCTAAAAAAAGGGTACCCGAC

>PP916604.1 Uncultured *Candidatus* Liberibacter sp. clone WTHC_02_Clas_Myagdi_Nepal_FWD 16S ribosomal RNA gene, partial sequence (>WTHC_02_Clas_Myagdi_Nepal_FWD)

AGGACACCGGTGGCGAAGGCGGCTCACTGGCCTGATACTGACGCTGAGGCGCGAAAGCGTGGGGAGC
AAACAGGATTAGATACCCTGGTAGTCCACGCCGTAAACGATGAGTGCTAGCTGTTGGGTGGTTTACCATT
CAGTGGCGCAGCTAACGCATTAAGCACTCCGCCTGGGGAGTACGGTCGCAAGATTAAAACTCAAAGGA
ATTGACGGGGGCCCGCACAAGCGGTGGAGCATGTGGTTTAATTCGATGCAACGCGCAGAACCTTACCA
GCCCTTGACATGTATAGGACGATATCAGAGATGGTATTTTCTTTTCGGAGACCTTTACACAGGTGCTGCAT
GGCTGTCGTCAGCTCGTGTCGTGAGATGTTGGGTTAAGTCCCGCAACGAGCGCAACCCCTGCCTCTAGT
TGCCATCAAGTTTAGGTTTTTACCTAATTGGGTTGGGTAAA

>PP916605.1 Uncultured *Candidatus* Liberibacter sp. clone WTHC_01_Clas_Palpa_Nepal_FWD 16S ribosomal RNA gene, partial sequence (>WTHC_01_Clas_Palpa_Nepal_FWD)

AGGACACCGGTGGCGAAGGCGGCTCACTGGCCTGATACTGACGCTGAGGCGCGAAAGCGTGGGGAGC
AAACAGGATTAGATACCCTGGTAGTCCACGCCGTAAACGATGAGTGCTAGCTGTTGGGTGGTTTACCATT
CAGTGGCGCAGCTAACGCATTAAGCACTCCGCCTGGGGAGTACGGTCGCAAGATTAAAACTCAAAGGA
ATTGACGGGGGCCCGCACAAGCGGTGGAGCATGTGGTTTAATTCGATGCAACGCGCAGAACCTTACCA
GCCCTTGACATGTATAGGACGATATCAGAGATGGTATTTTCTTTTCGGAGACCTTTACACAGGTGCTGCAT
GGCTGTCGTCAGCTCGTGTCGTGAGATGTTGGGTTAAGTCCCGCAACGAGCGCAACCCCTGCCTCTAGT
TGCCATCAAGTTTAGGTTTTTACCTAAAGGTTGGGGTAACCCAAC
