# Supplementary material for: PCR-based detection and phylogenetic analysis of Candidatus Liberibacter asiaticus in citrus orchards across Nepal
Source: PLoS One. 2026 May 27;21(5):e0333726. doi: 10.1371/journal.pone.0333726 (PMC13215486; doi:10.1371/journal.pone.0333726)
Supplement: S2 File — (PDF) [file pone.0333726.s002.pdf]

**Consensus sequence used in the construction of phylogenetic tree generated using SnapGene and AliView software.**

>PP916602.1 merged_PP916597.1 Dadeldhura_Nepal

ATTCCGAGTGTAGAGGTGAAATTCGTAGATATTCGGAGGAACACCGGTGGCGAAGGCGGCTCACTGGC
CTGATACTGACGCTGAGGCGCGAAAGCGTGGGGAGCAAACAGGATTAGATACCCTGGTAGTCCACGCC
GTAAACGATGAGTGCTAGCTGTTGGGTGGTTTACCATTCAGTGGCGCAGCTAACGCATTAAGCACTCCG
CCTGGGGAGTACGGTCGCAAGATTAAAACTCAAAGGAATTGACGGGGGCCCGCACAAGCGGTGGAGC
ATGTGGTTTAATTCGATGCAACGCGCAGAACCTTACCAGCCCTTGACATGTATAGGACGATATCAGAGAT
GGTATTTTCTTTTCGGAGACCTTTACACAGGTGCTGCATGGCTGTCGTCAGCTCGTGTCGTGAGATGTTG
GGTTAAGTCCCGCAACGAGCGCAACCCC

>PP916605.1 merged_PP916600.1 Palpa Nepal

ATTCCGAGTGTAGAGGTGAAATTCGTAGATATTCGGAGGAACACCGGTGGCGAAGGCGGCTCACTGGC
CTGATACTGACGCTGAGGCGCGAAAGCGTGGGGAGCAAACAGGATTAGATACCCTGGTAGTCCACGCC
GTAAACGATGAGTGCTAGCTGTTGGGTGGTTTACCATTCAGTGGCGCAGCTAACGCATTAAGCACTCCG
CCTGGGGAGTACGGTCGCAAGATTAAAACTCAAAGGAATTGACGGGGGCCCGCACAAGCGGTGGAGC
ATGTGGTTTAATTCGATGCAACGCGCAGAACCTTACCAGCCCTTGACATGTATAGGACGATATCAGAGAT
GGTATTTTCTTTTCGGAGACCTTTACACAGGTGCTGCATGGCTGTCGTCAGCTCGTGTCGTGAGATGTTG
GGTTAAGTCCCGCAACGAGCGCAACCCC

>PP916604.1 merged_PP916599.1 Myagdi_Nepal

ATTCCGAGTGTAGAGGTGAAATTCGTAGATATTCGGAGGAACACCGGTGGCGAAGGCGGCTCACTGGC
CTGATACTGACGCTGAGGCGCGAAAGCGTGGGGAGCAAACAGGATTAGATACCCTGGTAGTCCACGCC
GTAAACGATGAGTGCTAGCTGTTGGGTGGTTTACCATTCAGTGGCGCAGCTAACGCATTAAGCACTCCG
CCTGGGGAGTACGGTCGCAAGATTAAAACTCAAAGGAATTGACGGGGGCCCGCACAAGCGGTGGAGC
ATGTGGTTTAATTCGATGCAACGCGCAGAACCTTACCAGCCCTTGACATGTATAGGACGATATCAGAGAT
GGTATTTTCTTTTCGGAGACCTTTACACAGGTGCTGCATGGCTGTCGTCAGCTCGTGTCGTGAGATGTTG
GGTTAAGTCCCGCAACGAGCGCAACCCC

>PP916603.1 merged_PP916598.1 Dailekh_Nepal

ATTCCGAGTGTAGAGGTGAAATTCGTAGATATTCGGAGGAACACCGGTGGCGAAGGCGGCTCACTGGC
CTGATACTGACGCTGAGGCGCGAAAGCGTGGGGAGCAAACAGGATTAGATACCCTGGTAGTCCACGCC
GTAAACGATGAGTGCTAGCTGTTGGGTGGTTTACCATTCAGTGGCGCAGCTAACGCATTAAGCACTCCG
CCTGGGGAGTACGGTCGCAAGATTAAAACTCAAAGGAATTGACGGGGGCCCGCACAAGCGGTGGAGC
ATGTGGTTTAATTCGATGCAACGCGCAGAACCTTACCAGCCCTTGACATGTATAGGACGATATCAGAGAT
GGTATTTTCTTTTCGGAGACCTTTACACAGGTGCTGCATGGCTGTCGTCAGCTCGTGTCGTGAGATGTTG
GGTTAAGTCCCGCAACGAGCGCAACCCC

>PP916601.1 merged_PP916596.1 CaLas Kathmandu_Nepal

ATTCCGAGTGTAGAGGTGAAATTCGTAGATATTCGGAGGAACACCGGTGGCGAAGGCGGCTCACTGGC
CTGATACTGACGCTGAGGCGCGAAAGCGTGGGGAGCAAACAGGATTAGATACCCTGGTAGTCCACGCC
GTAAACGATGAGTGCTAGCTGTTGGGTGGTTTACCATTCAGTGGCGCAGCTAACGCATTAAGCACTCCG
CCTGGGGAGTACGGTCGCAAGATTAAAACTCAAAGGAATTGACGGGGGCCCGCACAAGCGGTGGAGC
ATGTGGTTTAATTCGATGCAACGCGCAGAACCTTACCAGCCCTTGACATGTATAGGACGATATCAGAGAT
GGTATTTTCTTTTCGGAGACCTTTACACAGGTGCTGCATGGCTGTCGTCAGCTCGTGTCGTGAGATGTTG
GGGTTAAGTCCCGCAACGGAGCGCAACCCC
