# Supplementary figures and images for: PCR-based detection and phylogenetic analysis of Candidatus Liberibacter asiaticus in citrus orchards across Nepal
Source: PLoS One. 2026 May 27;21(5):e0333726. doi: 10.1371/journal.pone.0333726 (PMC13215486; doi:10.1371/journal.pone.0333726)

**Original images for gel.**

Fig3a.tif

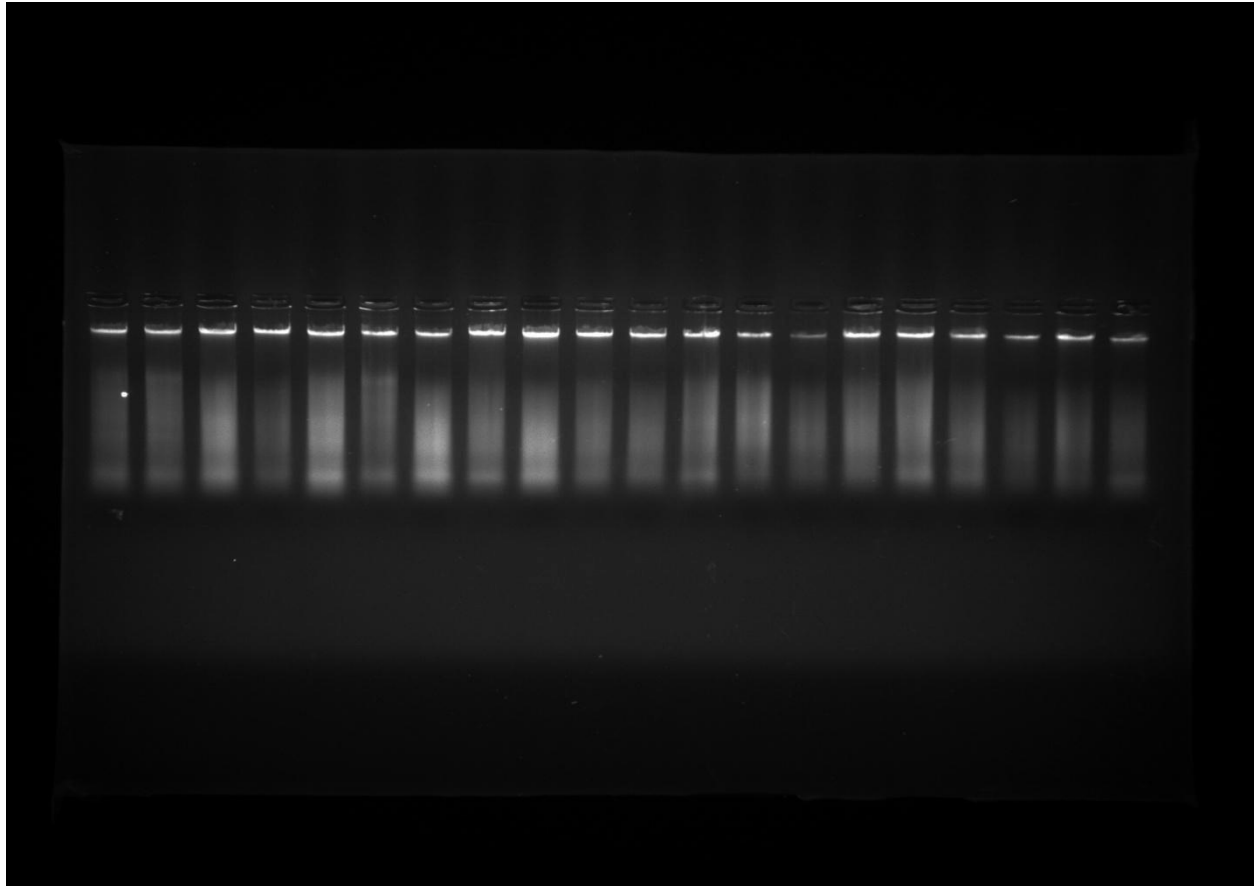

Fig3b.tif

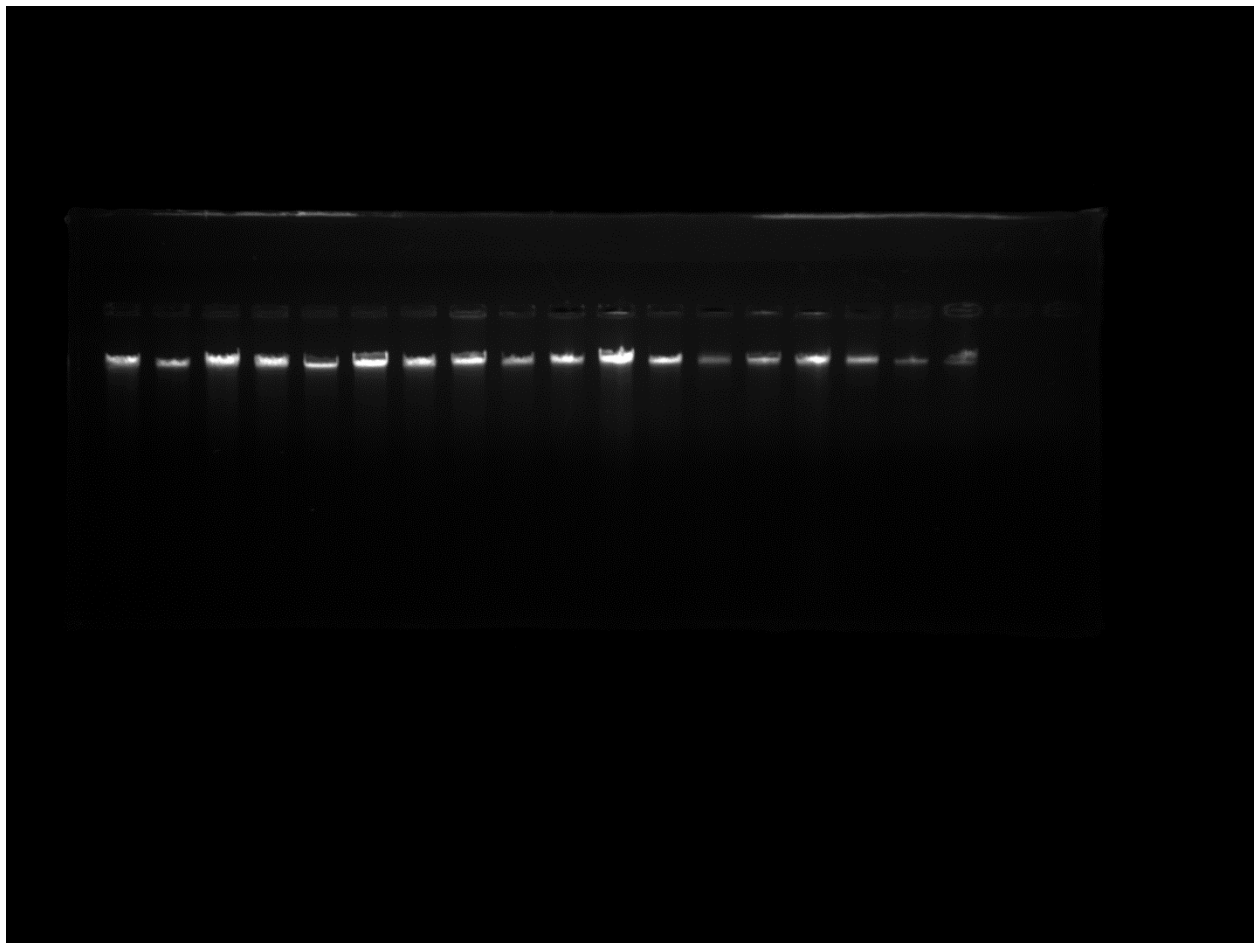

Fig4a.tif

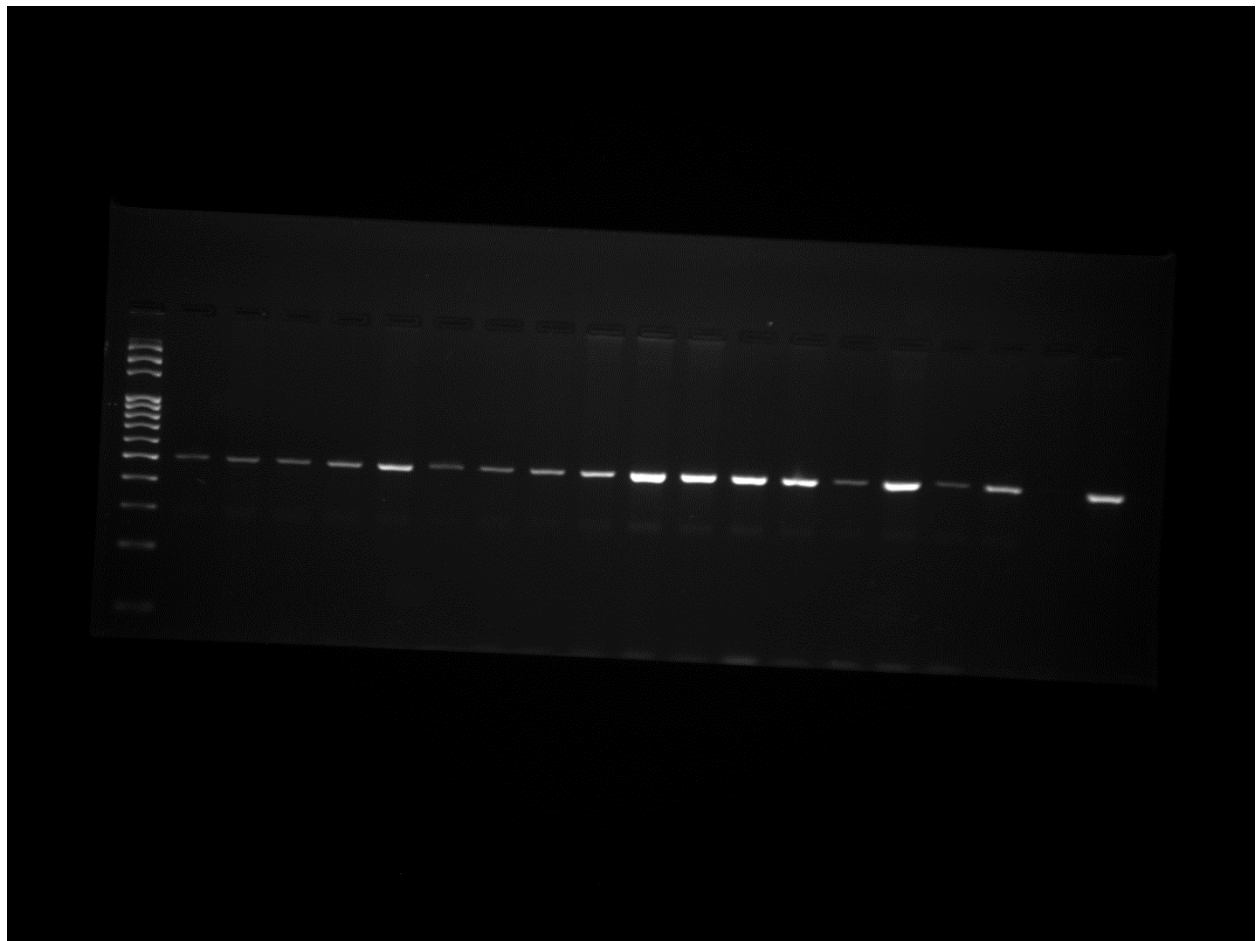

Fig4b.tif

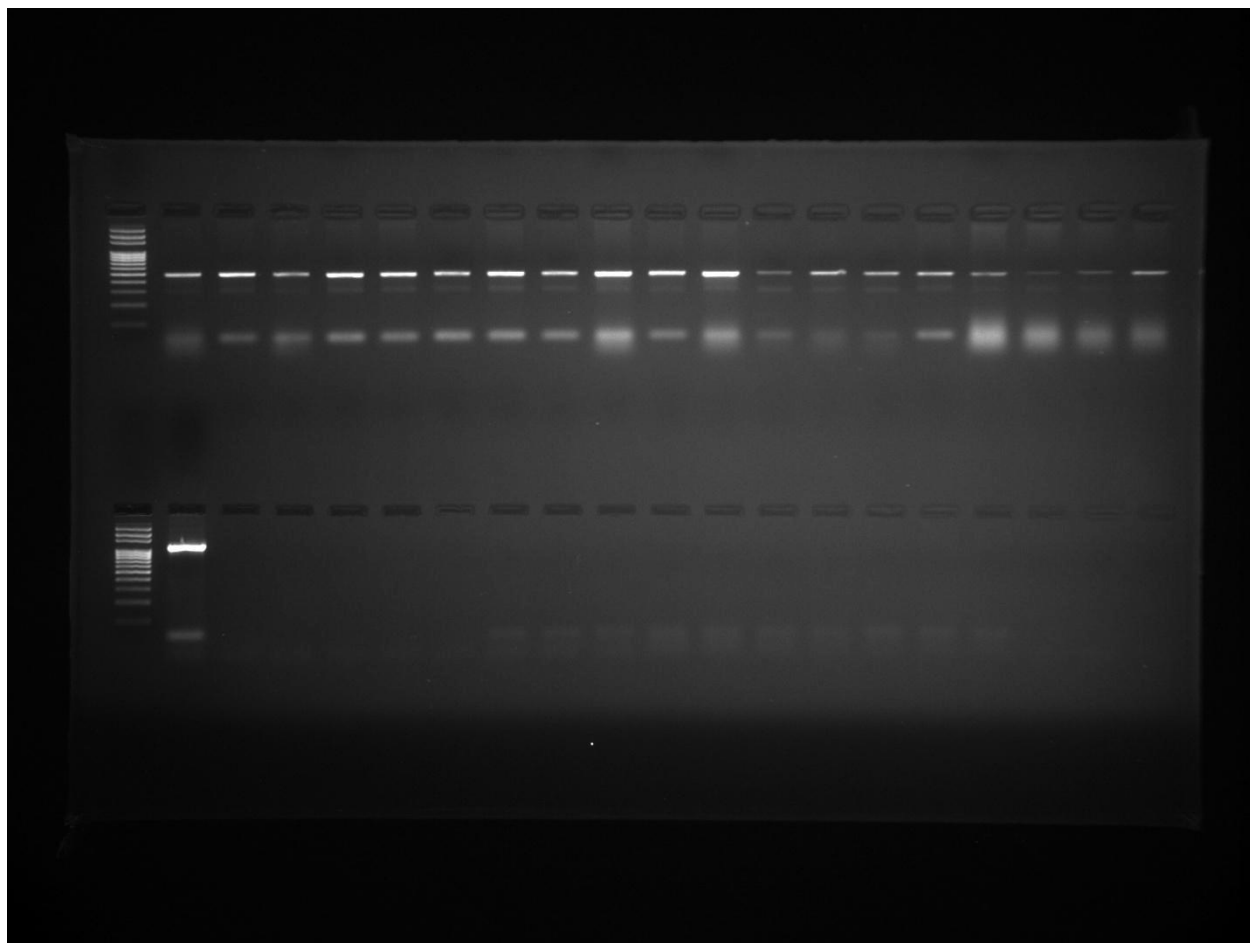

Fig4c.tif

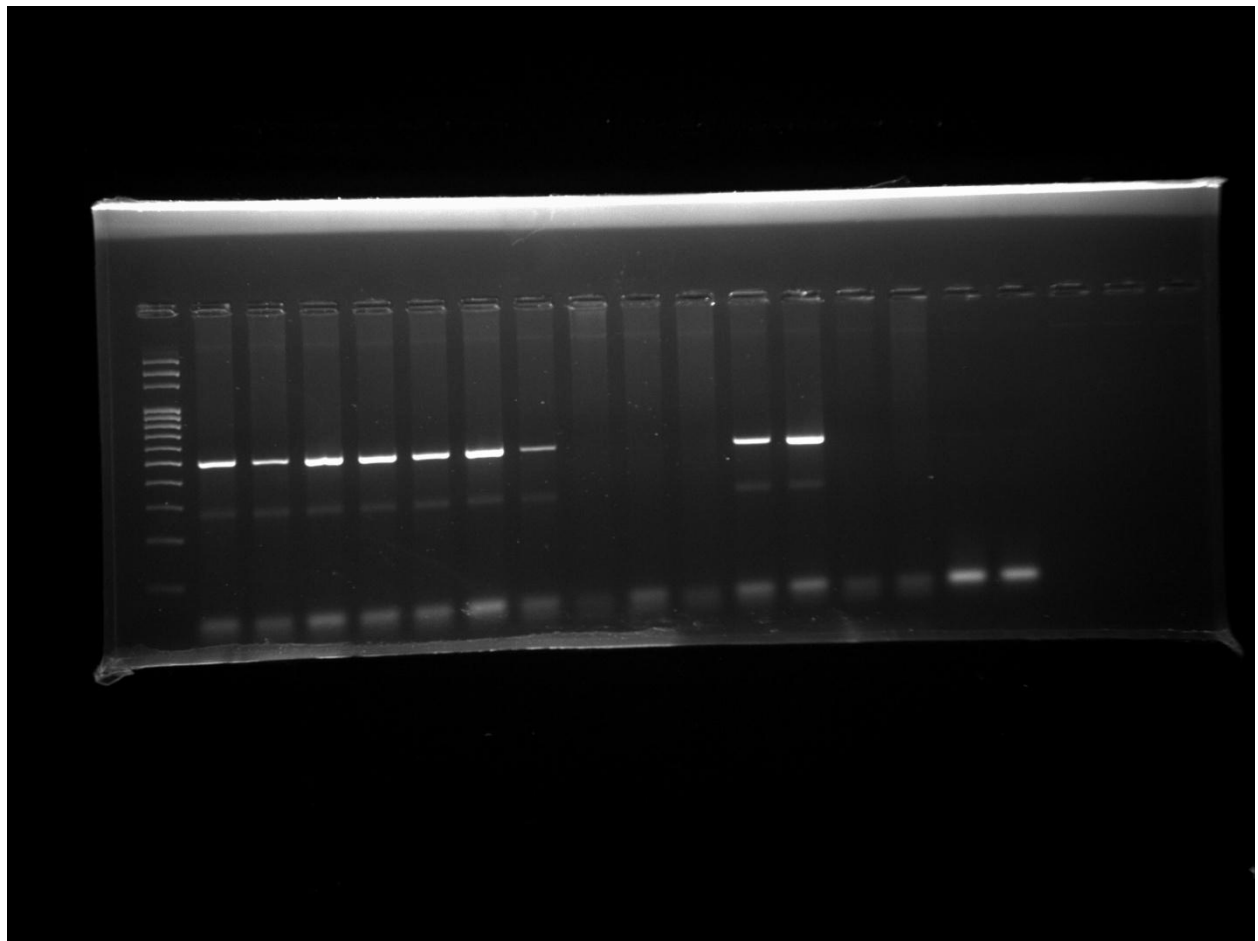

Supplement: S3 File — Original images for gel. (PDF) [file pone.0333726.s003.pdf]
