# Supplementary material for: PCR-based detection and phylogenetic analysis of Candidatus Liberibacter asiaticus in citrus orchards across Nepal
Source: PLoS One. 2026 May 27;21(5):e0333726. doi: 10.1371/journal.pone.0333726 (PMC13215486; doi:10.1371/journal.pone.0333726)
Supplement: Attachment — Submitted filename: Response to Reviewers.docx [file pone.0333726.s004.docx]

**1/19/2026**

**Dear Editor/ Reviewers,**
**We thank you and the reviewers for the valuable comments and suggestions on our manuscript titled “PCR-Based Detection and Phylogenetic Analysis of *Candidatus Liberibacter* *asiaticus* in Citrus Orchards Across Nepal” (Manuscript Number: PONE-D-25-50808R1). These inputs have helped us significantly improve the quality and clarity of our work. Below, we provide a point-by-point response to each comment. All changes have been incorporated in the revised manuscript accordingly.**

***Response to editor***

1. Please ensure that your manuscript meets PLOS ONE's style requirements, including those for file naming.

***Response****: We have revised the manuscript and all associated files to fully comply with PLOS ONE’s style and file-naming requirements*.

2. PLOS requires an ORCID iD for the corresponding author in Editorial Manager on papers submitted after December 6th, 2016. Please ensure that you have an ORCID iD and that it is validated in Editorial Manager. To do this, go to ‘Update my Information’ (in the upper left-hand corner of the main menu), and click on the Fetch/Validate link next to the ORCID field. This will take you to the ORCID site and allow you to create a new iD or authenticate a pre-existing iD in Editorial Manager.

- **Response**: ORCID iD for corresponding author Bal Kumari Oliya is <https://orcid.org/0000-0002-1908-5353> and is validated in Editorial Manager. Which is updated as suggested.

3. Thank you for stating the following in the Competing Interests section: [The authors have declared that no competing interests exist.].

We note that one or more of the authors are employed by a commercial company: Himalayan Plants Solution.

Please provide an amended Funding Statement declaring this commercial affiliation, as well as a statement regarding the Role of Funders in your study. If the funding organization did not play a role in the study design, data collection and analysis, decision to publish, or preparation of the manuscript and only provided financial support in the form of authors' salaries and/or research materials, please review your statements relating to the author contributions, and ensure you have specifically and accurately indicated the role(s) that these authors had in your study. You can update author roles in the Author Contributions section of the online submission form. Please also include the following statement within your amended Funding Statement. If your commercial affiliation did play a role in your study, please state and explain this role within your updated Funding Statement.

- **Response: "** **We thank the Editor for their comment.** The corresponding author Dr. Bal Kumari Oliya is the founder of Himalayan Plant Solution. The company did not pay in any form including salary to conduct this study. In addition, the company does not have any role in the study design, data collection and analysis, decision to publish, or preparation of the manuscript. The specific role of author is articulated in the ‘author contributions’ section.
- **Competing Interests Statement: We have removed the commercial company from the manuscript as this does not have any funding roles in this manuscript.** Therefore, in the updated file we have no competing interests to exist.
- **Author Contributions:** We have updated this section to use the required credit taxonomy, accurately detailing each author's specific intellectual contributions. The author’s contribution is as given below-
- **Funding Statement:** "This work was supported by in-kind contributions from the National Centre for Fruit Development (sample collection), the Warm Temperate Horticulture Centre (provision of reagents, laboratory workspace). The organization had no role in study design, data collection and analysis, decision to publish, or preparation of the manuscript.

1. Please also provide an updated Competing Interests Statement declaring this commercial affiliation along with any other relevant declarations relating to employment, consultancy, patents, products in development, or marketed products, etc.

Within your Competing Interests Statement, please confirm that this commercial affiliation does not alter your adherence to all PLOS ONE policies on sharing data and materials by including the following statement: "This does not alter our adherence to PLOS ONE policies on sharing data and materials.” (as detailed online in our guide for authors <http://journals.plos.org/plosone/s/competing-interests>) . If this adherence statement is not accurate and there are restrictions on sharing of data and/or materials, please state these. Please note that we cannot proceed with consideration of your article until this information has been declared.

Please include both an updated Funding Statement and Competing Interests Statement in your cover letter. We will change the online submission form on your behalf.

- Response: We have removed the commercial company from the author’s affiliation, as it does not have any funding support for this study.

4. Please amend the manuscript submission data (via Edit Submission) to include author Siddartha Gautam.

- **Response**: The submission data has been updated, and author Siddartha Gautam has now been added as requested.

5. Please amend your authorship list in your manuscript file to include author Siddartha Gautam Gautam.

- **Response**: We mistakenly entered the author’s name as “Siddartha Gautam” in the first name field and “Gautam” in the last name field, which resulted in the system displaying the name as “Siddartha Gautam Gautam.” The correct author’s name is **First name: Siddartha; Last name: Gautam**. There is no author named “Siddartha Gautam Gautam.”

6. We note that Figure 1 in your submission contains map images which may be copyrighted. All PLOS content is published under the Creative Commons Attribution License (CC BY 4.0), which means that the manuscript, images, and Supporting Information files will be freely available online, and any third party is permitted to access, download, copy, distribute, and use these materials in any way, even commercially, with proper attribution. For these reasons, we cannot publish previously copyrighted maps or satellite images created using proprietary data, such as Google software (Google Maps, Street View, and Earth). For more information, see our copyright guidelines: <http://journals.plos.org/plosone/s/licenses-and-copyright>.

We require you to either (1) present written permission from the copyright holder to publish these figures specifically under the CC BY 4.0 license, or (2) remove the figures from your submission:

1. You may seek permission from the original copyright holder of Figure 1 to publish the content specifically under the CC BY 4.0 license.

We recommend that you contact the original copyright holder with the Content Permission Form (<http://journals.plos.org/plosone/s/file?id=7c09/content-permission-form.pdf>) and the following text:

“I request permission for the open-access journal PLOS ONE to publish XXX under the Creative Commons Attribution License (CCAL) CC BY 4.0 (<http://creativecommons.org/licenses/by/4.0/>). Please be aware that this license allows unrestricted use and distribution, even commercially, by third parties. Please reply and provide explicit written permission to publish XXX under a CC BY license and complete the attached form.”

Please upload the completed Content Permission Form or other proof of granted permissions as an "Other" file with your submission.

In the figure caption of the copyrighted figure, please include the following text: “Reprinted from [ref] under a CC BY license, with permission from [name of publisher], original copyright [original copyright year].”

2. If you are unable to obtain permission from the original copyright holder to publish these figures under the CC BY 4.0 license or if the copyright holder’s requirements are incompatible with the CC BY 4.0 license, please either i) remove the figure or ii) supply a replacement figure that complies with the CC BY 4.0 license. Please check copyright information on all replacement figures and update the figure caption with source information. If applicable, please specify in the figure caption text when a figure is similar but not identical to the original image and is therefore for illustrative purposes only.

The following resources for replacing copyrighted map figures may be helpful:

USGS National Map Viewer (public domain): <http://viewer.nationalmap.gov/viewer/>
The Gateway to Astronaut Photography of Earth (public domain): <http://eol.jsc.nasa.gov/sseop/clickmap/>
Maps at the CIA (public domain): <https://www.cia.gov/library/publications/the-world-factbook/index.html> and [https://www.cia.gov/library/publications/cia-maps-publications/index.html](https://track.editorialmanager.com/CL0/https:%2F%2Fwww.cia.gov%2Flibrary%2Fpublications%2Fcia-maps-publications%2Findex.html/1/010f019a76a8ca9d-5381e136-a5f3-4f26-8241-2f150ef6795f-000000/HatcTwxxOvENW1pW3dxsGPr8CgOwHo2cPRf99_2Biho=237)
NASA Earth Observatory (public domain): <http://earthobservatory.nasa.gov/>
Landsat: <http://landsat.visibleearth.nasa.gov/>
USGS EROS (Earth Resources Observatory and Science (EROS) Center) (public domain): [http://eros.usgs.gov/#](http://eros.usgs.gov/)
Natural Earth (public domain): <http://www.naturalearthdata.com/>

- **Response**: The map used in Figure 1 was created by an external contributor who has released it under the Creative Commons Attribution–Share Alike 4.0 International (CC BY-SA 4.0) license. We confirm that the copyright holder has granted permission for reuse under this license, and the appropriate licensing statement has been added below

Link to the source:

URL: <https://commons.wikimedia.org/wiki/File:New_Map_of_Nepal_District_and_Province.svg>

7. PLOS ONE now requires that authors provide the original uncropped and unadjusted images underlying all blot or gel results reported in a submission’s figures or Supporting Information files. This policy and the journal’s other requirements for blot/gel reporting and figure preparation are described in detail at <https://journals.plos.org/plosone/s/figures#loc-blot-and-gel-reporting-requirements> and <https://journals.plos.org/plosone/s/figures#loc-preparing-figures-from-image-files>. When you submit your revised manuscript, please ensure that your figures adhere fully to these guidelines and provide the original underlying images for all blot or gel data reported in your submission. See the following link for instructions on providing the original image data: <https://journals.plos.org/plosone/s/figures#loc-original-images-for-blots-and-gels>.

In your cover letter, please note whether your blot/gel image data are in Supporting Information or posted at a public data repository, provide the repository URL if relevant, and provide specific details as to which raw blot/gel images, if any, are not available. Email us at [plosone@plos.org](mailto:plosone@plos.org) if you have any questions.

- **Response**: We have revised the figures in accordance with the PLOS ONE blot/gel reporting guidelines. The original, uncropped, and unadjusted gel images underlying all reported results have now been provided as Supporting Information, as required.

8. Please upload a new copy of Figure 6 as the detail is not clear. Please follow the link for more information: <https://journals.plos.org/plosone/s/figures>

***Response****: A new, high-quality version of Figure 6 has been uploaded as requested. The issue has been addressed.*

***Response to reviewers***

1. Is the manuscript technically sound, and do the data support the conclusions?

The manuscript must describe a technically sound piece of scientific research with data that supports the conclusions. Experiments must have been conducted rigorously, with appropriate controls, replication, and sample sizes. The conclusions must be drawn appropriately based on the data presented.

Reviewer #1: Yes

Reviewer #2: Partly

***Response****: We confirm that the manuscript presents technically sound research with appropriate controls, replication, and sample sizes, and that all conclusions are fully supported by the data.*

2. Has the statistical analysis been performed appropriately and rigorously?

Reviewer #1: Yes

Reviewer #2: I Don't Know

***Response****: Yes, the statistical analysis has been performed appropriately and rigorously.*

3. Have the authors made all data underlying the findings in their manuscript fully available?

The [PLOS Data policy](http://track.editorialmanager.com/CL0/http:%2F%2Fwww.plosone.org%2Fstatic%2Fpolicies.action%23sharing/1/010f019a76a8ca9d-5381e136-a5f3-4f26-8241-2f150ef6795f-000000/DuvjbWDz5YD-6bmfEFzNPkHktJ8723w9JCj18R6UZo8=237) requires authors to make all data underlying the findings described in their manuscript fully available without restriction, with rare exception (please refer to the Data Availability Statement in the manuscript PDF file). The data should be provided as part of the manuscript or its supporting information, or deposited to a public repository. For example, in addition to summary statistics, the data points behind means, medians and variance measures should be available. If there are restrictions on publicly sharing data—e.g. participant privacy or use of data from a third party—those must be specified.

Reviewer #1: Yes

Reviewer #2: Yes

***Response****: Yes, all underlying data have been fully provided as required.*

4. Is the manuscript presented in an intelligible fashion and written in standard English?

PLOS ONE does not copyedit accepted manuscripts, so the language in submitted articles must be clear, correct, and unambiguous. Any typographical or grammatical errors should be corrected at revision, so please note any specific errors here.

Reviewer #1: Yes

Reviewer #2: Yes

***Response****: Yes, the manuscript is presented in an intelligible fashion and written in standard English.*

5. Review Comments to the Author

Please use the space provided to explain your answers to the questions above. You may also include additional comments for the author, including concerns about dual publication, research ethics, or publication ethics. (Please upload your review as an attachment if it exceeds 20,000 characters)

Reviewer #1: The paper found satisfactory after my review. The author has done all the requisites related to the title mentioned. The results are highly correlated. Research work is highly appreciated. Data produced is genuine.

Reviewer #2: Lack of background depth: The study briefly mentions the global significance of citrus greening but does not explicitly state the current status or prior studies in Nepal. How this study builds upon or differs from existing regional work would strengthen its significance.
Limited sequencing depth: Only five samples were subjected to Sanger sequencing, which is insufficient for strong conclusions on genetic diversity.

Response

- We thank the reviewer for this valuable suggestion to improve the context and significance of our study. We agree that a clearer background is essential. We have critically revised the manuscript. There is limited data published from Nepal on the diagnosis of this disease. Although several review articles exist, research generating primary data remains scarce. Only a few studies on citrus greening have been reported, and the PCR-based diagnostic study by Oliya et al. (2024) was already included in the manuscript. In the revised version, we have incorporated additional information in context of Nepal. Diagnosis of Citrus Greening by Oliya (2014), PCR-based HLB diagnosis by Chhetri et al. (2019), and the field-level scratch test by Khadka et al. (2010) are added. The research presented in these papers is quite limited when compared with this study, which provides one of the most comprehensive PCR-based assessments of citrus greening in Nepal. Therefore, this research provides a strong foundation for future disease surveillance and HLB management, especially in the context of the limited large-scale diagnostic information available so far. To explicitly address this gap, the last paragraph of the introduction is written as-

“In Nepal, HLB was first reported in 1967 in the Pokhara Valley [19]. Since then, the disease has been rapidly observed in different parts of the country, leading to widespread citrus decline [6,17,18,20–22]. Many studies on HLB in Nepal have largely relied on surveys and visual assessment [18,22], with only a few employing conventional PCR for confirmation [6,17,59,60] . Therefore, the present research aims to systematically detect Ca. L. asiaticus in suspected citrus trees from diverse citrus-growing regions of Nepal using conventional PCR with the Las606/LSS primer set, followed by Sanger sequencing. This approach will provide the comprehensive molecular data necessary to investigate the origin and phylogenetic relationships of the pathogen, both within Nepal and in a global context.”.

**Our study builds upon and differs from existing regional work:**

- **From Detection to Characterization:** While prior Nepalese studies stopped at visual assessment or basic PCR detection, our work advances to **molecular characterization and sequencing**, generating the first Nepalese *C. L. asiaticus* as sequences (GenBank PP916596–PP916605).
- **Enabling Comparative Phylogenetics:** For the first time, this allows the Nepalese pathogen population to be analyzed in a **regional phylogenetic context** alongside sequences from India, China, and beyond, which we now address in the Discussion.
- **Foundational Data Creation:** Our study moves the field from observational records to creating a **public, foundational molecular dataset** upon which future diversity studies, diagnostic tool development, and management strategies for Nepal can be built, we have addressed in last paragraph of discussion and conclusion in the revised manuscript.
- **Limited sequencing depth:** We thank the reviewer for raising this critical point regarding sequencing depth. We acknowledge that data from five samples cannot delineate the full spectrum of *C. L. asiaticus* genetic diversity in Nepal.

We wish to clarify the primary objective and context of our study:

- **Primary Objective:** Our goal was not a comprehensive population diversity study, but to achieve the **molecular confirmation and initial genetic characterization** of *C. L. asiaticus* in Nepal, which we have included in the revised manuscript.
- **Strategic Sample Selection:** The five samples sequenced were carefully selected as representatives from five geographically diverse, major citrus-growing provinces (Karnali, Gandaki, Sudur Paschim, Lumbini and Bagmati), providing a strategically chosen first snapshot.
- **Rigorous Methodology:** We performed forward and reverse sequencing for high-confidence base calls, applied multiple bioinformatics tools (SnapGene, AliView, MEGA), and have deposited all 10 raw sequences in NCBI GenBank (PP916596–PP916605) to ensure transparency and utility for future research.

We have taken the reviewer's feedback seriously and have **added possible limitations it may have in the discussion of the revised manuscript.**

. Reviewer #2**: No details on PCR validation: The abstract does not mention whether positive and negative controls, sensitivity checks, or replicates were used to confirm PCR reliability.**

**Response**:

- We thank the reviewer for this important observation. We acknowledge that these critical methodological details for ensuring PCR reliability were omitted from the original abstract. As stated in the Materials and Methods. In this study, the reliability of PCR was ensured by including previously confirmed positive and negative control samples in every PCR cycle. Furthermore, each assay was performed in triplicate to guarantee consistency and reproducibility of the results. These measures confirm the accuracy and reliability of our PCR data, and this information has now been incorporated into the revised manuscript

Reviewer #2**: No** environmental or management correlation: The study does not discuss about the factors like altitude, climate, or management practices that might influence disease spread.

**Response:**

- We sincerely thank the reviewer for raising this critical point. We agree that disease epidemiology is driven by a combination of pathogen biology, environmental factors, and management practices. To address this, we have expanded the discussion to explicitly link our findings of limited genetic diversity to sampling from areas with similar climatic conditions like temperature and altitude. We now state that disease spread may be attributed to grafting with uncertified rootstocks and scions, as well as the improper management of citrus orchards. The Conclusion now synthesizes key risk factors: the lack of effective management practices, the use of uncertified planting materials, relatively uniform mid hill climatic condition, and the limited vector management.

Reviewer #2**: The abstract could better link the molecular findings to practical outcomes for farmers or policy.**

**Response:**

- We sincerely thank the reviewer for raising this critical point. The molecular findings are now explicitly linked to practical outcomes in the revised abstract. We demonstrate their relevance by stating how the confirmation of *Candidatus Liberibacter asiaticus* in Nepal: 1) provides the critical genetic baseline needed for developing region-specific diagnostic tools, 2) informs surveillance strategies by confirming the pathogen's presence, and 3) highlights the urgent need for policy to support management practices such as using clean planting material and vector control. This directly guides farmer actions and policy decisions for HLB control.".

Reviewer #2**: Future management or diagnostic development prospects are mentioned vaguely without specific pathways**.

***Response***:

- We are grateful to the reviewer for their insightful comments, which have greatly strengthened the manuscript. We have addressed both concerns with substantial revisions to the Discussion and Conclusion. We have replaced vague prospects with concrete recommendations. We critically evaluate the limitations of conventional PCR and propose a specific pathway: “Nepal should consider developing a real-time PCR-based detection system," citing internationally validated EPPO protocols [70]. We advocate for the immediate adoption of the highly sensitive **Las606/LSS primer set** for its robustness with field samples. Finally, we define clear management policies: "the use of certified clean planting material and vector psyllid control programs."

Reviewer #2: Time duration to reach the laboratory from collection sites and locations from where the samples have been collected are not mentioned.

***Response***:

- Information on the time taken to transport samples to the laboratory and the specific locations from which they were collected has been added to the Methodology section of the revised manuscript. **This addition ensures transparency and allows for the assessment of potential sample degradation during transit.**

**References**

6. Oliya BK. Diagnosis of citrus greening (huanglongbing) disease by PCR and citrus tresteza virus by DAS-ELISA techniques from different parts of Nepal. Dissertation, Tribhuvan University. 2014.

17.	Oliya B, Giri R, Sharma S, Bhandari S, Karki S, Adhikari P, et al. *Candidatus liberibacter asiaticus*" associated with mandarin orange (*Citrus reticulata* Blanco) decline in Nepal. In: Karki S, Poudel P, Shrestha Y, Baral S, editors. Mandarin orange: history, science and technology in Nepal. Kathmandu: NHS and NCFD; 2024. pp. 299–305.

18.	Garnier M, Bové JM. Distribution of the Huanglongbing (Greening) *Liberobacter* species in fifteen African and Asian countries. International organization of citrus virologists conference proceedings (1957-2010). 1996;13. doi:10.5070/C58V467619

19. Knorr LC, Shah SM, Gupta OP. Greening disease of citrus in Nepal. Plant Dis Reporter. 1970;54: 1092–1095.

20. Kandel BP, Chhetri LB. Effect of integrated plant nutrient management system in quality of mandarin orange (*Citrus reticulata* Blanco). Jurnal Agercolere. 2019;1: 19–24. doi:10.37195/jac.v1i1.54

21.	Acharya UK, Adhikari D. Strategies for managing citrus decline in Nepal. Advancing horticulture for fallow land utilization and commercialization. Kirtipur: NHS; 2013. pp. 35–43.

22.	Paudel RC, Regmi C, Shrestha S, Rana N, Sijapati J, Maharjan J, et al. Scale of huanglongbing disease infection and the status of molecular diagnostic tests of mandarin orange groves in Nepal. Mandarin Orange: History, Science and Technology in Nepal. Kathmandu: NHS and NCFD; 2024. pp. 361–377.

59. Chhetri SB, Shrestha M, Shrestha S. Polymerase Chain Reaction (PCR) - Based Diagnosis of Huanglongbing (Citrus Greening) Disease in Salyan District of Nepal. Journal of Agriculture and Environment. 2019;20: 57–66. doi:10.3126/aej.v20i0.25010

60. Jagoueix S, Bové JM, Garnier M. PCR detection of the two *Candidatus liberobacter* species associated with greening disease of citrus. Mol Cell Probes. 1996;10: 43–50. doi:10.1006/mcpr.1996.0006

53. Shweta, Singh J, Kashyap P, Kumar P, Kumar R, Panwar AS, et al. Molecular identification of citrus greening bacterium associated with Kinnow Mandarin in Western Uttar Pradesh, India. Indian Phytopathol. 2021;74: 1135–1141. doi:10.1007/s42360-021-00396-2

54.	Ding F, Deng X, Hong N, Zhong Y, Wang G, Yi G. Phylogenetic analysis of the citrus Huanglongbing (HLB) bacterium based on the sequences of 16S rDNA and 16S/23S rDNA intergenic regions among isolates in China. Eur J Plant Pathol. 2009;124: 495–503. doi:10.1007/s10658-009-9436-0

55.	Zheng Y, Li J, Zheng M, Li Y, Deng X, Zheng Z. Whole genome sequences of 135 “Candidatus Liberibacter asiaticus” strains from China. Sci Data. 2024;11: 1018. doi:10.1038/s41597-024-03855-3

70. de Chaves MQ-G, Morán F, Barbé S, Bertolini E, de la Rosa FS, Marco-Noales E. A new and accurate qPCR protocol to detect plant pathogenic bacteria of the genus ‘Candidatus Liberibacter’ in plants and insects. Sci Rep. 2023;13: 3338. doi:10.1038/s41598-023-30345-0
