# Supplementary material for: PCR-based detection and phylogenetic analysis of Candidatus Liberibacter asiaticus in citrus orchards across Nepal
Source: PLoS One. 2026 May 27;21(5):e0333726. doi: 10.1371/journal.pone.0333726 (PMC13215486; doi:10.1371/journal.pone.0333726)
Supplement: Attachment — Submitted filename: Comments to the Author.docx [file pone.0333726.s005.docx]

**Comments to the Author**

The author addresses an important phytopathological problem (HLB) and provides country-wide molecular detection data from Nepal, which has regional significance. The dataset 1026 samples is strong, and the study attempts both diagnostics and phylogenetics. However, scientific depth, methodological rigor are insufficient for PLOS ONE in the current form. Several major conceptual and technical limitations must be addressed.

**Major comments**

Study primarily relies on conventional PCR and 16S rRNA sequencing, which are standard approaches. The claim of being a “comprehensive genetic characterization” is overstated given the limited depth of analysis.

Out of 1026 samples, only five were sequenced without justification of selection criteria. This is insufficient to represent genetic diversity across Nepal.

Phylogeny is based only on a short 16S rRNA fragment (~500 bp), which lacks resolution for intra-species variation. Use of Neighbor-Joining further weakens evolutionary interpretation.

“Candidatus” should be italicized, while “Liberibacter asiaticus” should not be italicized as currently presented. Please ensure consistent and correct formatting of this nomenclature throughout the manuscript.

References are not formatted according to the journal’s guidelines. Please revise all citations and the reference list to strictly comply with the required journal format.

Manuscript lacks essential tables. It is recommended to include tables for primer details, criteria for sample selection for sequencing, and a summary of accession numbers to improve clarity and organization.

**Minor Comments**

**Line 24–25**: “Ca. L. asiaticus (Ca. L. asiaticus)” → redundant repetition.

**Line 39–40**: Sentence is repetitive and can be shortened for clarity.

**Line 58**: “Gram-negative bacteria Candidatus Liberibacter spp.” → should be “Gram-negative bacterium” or restructure sentence.

**Line 208**: “25-well thermal cycler” → incorrect terminology; should specify PCR machine model.

**Line 209–211**: PCR reaction volumes do not sum correctly to 15 µl; needs correction.

**Line 370**: Missing space before citation “[41]”

**Line 323–324**: “citrus tresteza virus” → spelling error (tristeza).

**Conclusion section**: Repeats statements from abstract; should be more concise and focused on implications.

**Please Cite these below references**

Bharsakale, R. D., Alex, B. K., Gubyad, M. G., Kokane, A. D., Kokane, S. B., Shukla, P. K., & Ghosh, D. K. (2025). Development of a novel duplex PCR-lateral flow immunoassay (d-PCR-LFIA) for simultaneous detection of Candidatus Liberibacter asiaticus and citrus tristeza virus. *Tropical Plant Pathology*, *50*(1), 77.

Ghosh, D., Kokane, S., Savita, B. K., Kumar, P., Sharma, A. K., Ozcan, A., ... & Santra, S. (2022). Huanglongbing pandemic: current challenges and emerging management strategies. *Plants*, *12*(1), 160.

**For the listed above comments, I would recommend to the editor accept with major revisions for this manuscript.**
