# Supplementary material for: PCR-based detection and phylogenetic analysis of Candidatus Liberibacter asiaticus in citrus orchards across Nepal
Source: PLoS One. 2026 May 27;21(5):e0333726. doi: 10.1371/journal.pone.0333726 (PMC13215486; doi:10.1371/journal.pone.0333726)
Supplement: Attachment — Submitted filename: Response_to_Reviewers_auresp_2.docx [file pone.0333726.s006.docx]

**[Date]**

**Dear Editor/ Reviewers,**
**We thank you and the reviewers for the valuable comments and suggestions on our manuscript titled “PCR-Based Detection and Phylogenetic Analysis of *Candidatus* Liberibacter asiaticus in Citrus Orchards Across Nepal” (Manuscript Number: PONE-D-25-50808R2). These inputs have helped us significantly improve the quality and clarity of our work. Below, we provide a point-by-point response to each comment. All changes have been incorporated in the revised manuscript accordingly.**

***Reviewer #3: Comments to the Author***

**The author addresses an important phytopathological problem (HLB) and provides country-wide molecular detection data from Nepal, which has regional significance. The dataset 1026 samples is strong, and the study attempts both diagnostics and phylogenetics. However, scientific depth, methodological rigor are insufficient for PLOS ONE in the current form. Several major conceptual and technical limitations must be addressed.**

**Major comments
Study primarily relies on conventional PCR and 16S rRNA sequencing, which are standard approaches. The claim of being a “comprehensive genetic characterization” is overstated given the limited depth of analysis.**

*Response: We agree with the concern raised and have removed the term “comprehensive genetic characterization” from the manuscript. The text has been revised to more accurately reflect the scope and depth of the analysis based on conventional PCR and 16S rRNA sequencing*.

**Out of 1026 samples, only five were sequenced without justification of selection criteria. This is insufficient to represent genetic diversity across Nepal.**

*Response: We acknowledge that data from five samples cannot delineate the full spectrum of C.* L. asiaticus *genetic diversity in Nepal*.

*We wish to clarify the primary objective and context of our study:*

- ***Primary Objective:*** *Our goal was not a comprehensive population diversity study, but to achieve the* ***molecular confirmation and initial genetic characterization*** *of C.* L. asiaticus *in Nepal, which we have included in the revised manuscript.*
- ***Strategic Sample Selection:*** *The five samples sequenced were carefully selected as representatives from* *five geographically diverse,* *major citrus-growing provinces (Karnali, Gandaki, Sudur Paschim, Lumbini and Bagmati), providing a strategically chosen first snapshot.*
- ***Rigorous Methodology:*** *We performed forward and reverse sequencing for high-confidence base calls, applied multiple bioinformatics tools (SnapGene, AliView, MEGA), and have deposited all 10 raw sequences in NCBI GenBank (PP916596–PP916605) to ensure transparency and utility for future research.*

*The manuscript now provides a justification for the selection of the five sequenced samples. We have also removed references to “genetic diversity” and focused the text on sequence analysis.*

**Phylogeny is based only on a short 16S rRNA fragment (~500 bp), which lacks resolution for intra-species variation. Use of Neighbor-Joining further weakens evolutionary interpretation.**

*Response:* *We thank the reviewer for this insightful comment. In the revised manuscript, we have used the Maximum Likelihood (ML) method with 500 bootstrap replicates to perform evolutionary analysis of the sequences. The ML method applies statistical models of sequence evolution to infer the tree that best fits the data, providing a more robust evolutionary interpretation compared to distance-based methods like Neighbor-Joining method. We have taken the reviewer's feedback seriously and have* ***added possible limitations it may have in the discussion of the revised manuscript****.* ***Fig 6*** *has now been revised.*

**“Candidatus” should be italicized, while “Liberibacter asiaticus” should not be italicized as currently presented. Please ensure consistent and correct formatting of this nomenclature throughout the manuscript.**

*Response: The formatting of Candidatus* Liberibacter asiaticus *has been corrected and standardized consistently throughout the manuscript.*

**References are not formatted according to the journal’s guidelines. Please revise all citations and the reference list to strictly comply with the required journal format.**

*Response: We have carefully revised all citations and the reference list to ensure full compliance with the journal’s formatting guidelines.*

**Manuscript lacks essential tables. It is recommended to include tables for primer details, criteria for sample selection for sequencing, and a summary of accession numbers to improve clarity and organization.**

*Response: We appreciate the reviewer’s suggestion. Tables detailing primer sequences, criteria for sample selection for sequencing and a summary of accession numbers have now been added to the manuscript to improve clarity and organization.*

**Minor Comments**
**Line 24–25: “Ca. L. asiaticus (Ca. L. asiaticus)” → redundant repetition.**

*Response: The redundant repetition has been corrected in the revised manuscript.*

**Line 39–40: Sentence is repetitive and can be shortened for clarity.**

*Response: The sentence has been revised and shortened to improve clarity.*
**Line 58: “Gram-negative bacteria Candidatus Liberibacter spp.” → should be “Gram-negative bacterium” or restructure sentence.**

*Response: The sentence has been revised to correct the grammatical inconsistency.*
**Line 208: “25-well thermal cycler” → incorrect terminology; should specify PCR machine model.**

*Response: The description has been revised to specify the PCR machine (Techne TC-312 thermal cycler) model accurately.*
**Line 209–211: PCR reaction volumes do not sum correctly to 15 µl; needs correction.**

*Response: The PCR reaction volumes have been corrected.*
**Line 370: Missing space before citation “[41]”**

*Response: The missing space before the citation has been corrected in the revised manuscript.*
**Line 323–324: “citrus tresteza virus” → spelling error (tristeza).**

*Response: The spelling of “citrus tristeza virus” has been corrected.*
**Conclusion section: Repeats statements from abstract; should be more concise and focused on implications**.

Response: The Conclusion section has been revised to be more concise and focused on the study’s key implications.

**Addition of paper**

**Following papers have been cited in the revised manuscript**

11. Bharsakale RD, Alex BK, Gubyad MG, Kokane AD, Kokane SB, Shukla PK, et al. Development of a novel duplex PCR-lateral flow immunoassay (d-PCR-LFIA) for simultaneous detection of *Candidatus* Liberibacter asiaticus and citrus tristeza virus. Trop Plant Pathol. 2025;50: 77. doi: 10.1007/s40858-025-00766-5.

24. Ghosh D, Kokane S, Savita BK, Kumar P, Sharma AK, Ozcan A, et al. Huanglongbing Pandemic: Current Challenges and Emerging Management Strategies. Plants. 2022;12(1): 160. doi:10.3390/plants12010160.
